# Enhanced Risk of Gastroesophageal Reflux Disease and Esophageal Complications in the Ulcerative Colitis Population

**DOI:** 10.3390/jcm13164783

**Published:** 2024-08-14

**Authors:** Xiaoliang Wang, Omar Almetwali, Jiayan Wang, Zachary Wright, Eva D. Patton-Tackett, Stephen Roy, Lei Tu, Gengqing Song

**Affiliations:** 1 Gastroenterology, Hepatology & Nutrition, Digestive Disease & Surgery Institute, Cleveland Clinic Main Campus, Cleveland, OH 44195, USA; wangxi@marshall.edu; 2 Joan C. Edwards School of Medicine, Marshall University Internal Medicine, Huntington, WV 25701, USA; almetwali@marshall.edu (O.A.); wangji@marshall.edu (J.W.); wright476@marshall.edu (Z.W.); pattont@marshall.edu (E.D.P.-T.); roy31@marshall.edu (S.R.); 3 Division of Gastroenterology, Union Hospital, Tongji Medical College, Huazhong University of Science and Technology, Wuhan 430022, China; 4 Department of Gastroenterology and Hepatology, Metrohealth Medical Center, Case Western Reserve University, Cleveland, OH 44109, USA

**Keywords:** ulcerative colitis, gastroesophageal reflux disease [GERD], Barrett’s esophagus, esophageal stricture

## Abstract

**Background**: Although heartburn and reflux are frequently reported in ulcerative colitis [UC], the correlation between UC and gastroesophageal reflux disease [GERD], and its complications, esophageal stricture and Barrett’s esophagus [BE], is not well understood. This study aims to examine the prevalence and associated risk of GERD and its complications within the UC population. **Methods**: We analyzed the National Inpatient Sample (NIS) dataset, consisting of 7,159,694 patients, comparing GERD patients with and without UC to those without GERD. We assessed the degree of colonic involvement in UC and the occurrence of esophageal complications. Bivariate analyses were conducted using the chi-squared test or Fisher exact test (two-tailed). **Results**: A higher prevalence of GERD (23.0% vs. 16.5%) and GERD phenotypes, such as non-erosive reflux disease (NERD) (22.3% vs. 16%) and erosive esophagitis (EE) (1.2% vs. 0.6%), was found in UC patients (*p* < 0.01), including pancolitis, proctitis, proctosigmoiditis, left-sided colitis, and indetermined UC (with undefined colonic involvement). UC patients were more likely to develop GERD (1.421), NERD (1.407), and EE (1.681) (*p* < 0.01). A higher prevalence of esophageal stricture (16.9 vs. 11.4 per 10,000 patients) and BE without dysplasia (94.5 vs. 39.3 per 10,000 patients) was found in UC (*p* < 0.05). The odds of developing BE without dysplasia were higher (1.892) in patients with UC (*p* < 0.01), including ulcerative pancolitis, proctitis, and indeterminate UC (OR of 1.657, 3.328, and 1.996, respectively) (*p* < 0.05). **Conclusions**: Our study demonstrates an increased risk of developing GERD and its complications in UC. This highlights the importance of vigilant monitoring and early intervention to minimize associated GERD-related risks in patients with UC.

## 1. Introduction

Inflammatory bowel disease (IBD) is typically classified into the following types: Crohn’s disease, ulcerative colitis (UC), and intermediate colitis (also referred to as indeterminant colitis). This classification is based on the specific regions of the gastrointestinal tract that are affected and the distinct histopathological features associated with each condition [1]. UC predominantly affects the colon in a continuous submucosal and mucosal inflammatory pattern with crypt abscesses. Clinically, UC presents with symptoms of diarrhea, fatigue, weight loss, lower gastrointestinal bleeding, and abdominal pain. UC is diagnosed by histopathological sampling from intestinal mucosa retrieved by means of endoscopic intervention with biopsy [1,2,3]. The prevalence of UC has seen a significant rise globally, with both developed and developing countries experiencing an increase in the number of cases [4,5]. While the exact causes of this increase remain unclear, several potential predisposing factors have been elected as potential culprits for this recent trend, including genetic predisposition, environmental triggers, changes in lifestyle, dietary habits, and alterations in the gut microbiota [6,7]. Genetic factors play a crucial role in the development of UC. Studies have identified several genetic variants associated with an increased risk of developing the disease [8,9]. Individuals with a family history of UC are at a higher risk, underscoring a potential hereditary process. However, it is important to note that not all individuals with a genetic predisposition will develop UC, highlighting the potential underlying role of other contributing factors [8]. Given the heterogenous nature of IBD phenotypes, genetic determinants of disease phenotypes play a crucial role in early diagnoses and a tailored treatment approach. Some studies have proposed using a Polygenic Risk Score approach (PRS) derived from Genome-Wide Association studies (GWASs). These studies identify genetic variants associated with a disease process by comparing a large set of individuals with a disorder to a large set of individuals without the disorder. These tools were further evaluated for their role in identifying and then aggregating several genetic variants in predicting lifetime risk of UC. GWASs uncovered over 240 independent genetic susceptibility loci for IBD [10,11,12], echoed by a seemingly genetically derived increased susceptibility of patients with autoimmune mediated diseases such as Ankylosing Spondylitis and Coeliac disease to develop IBD [13,14]. However, while these studies highlighted the potential genetic component in disease predisposition, these risk scores are far from being employed clinically as tools predictive of disease risk as they require patient population-specific tailoring [15,16,17]. Moreover, the role of the HLA complex (also referred to as the Major Histocompatibility Complex [MHC], located on chromosome 6) has been extensively investigated. Despite its well-established influence on chronic inflammatory diseases by means of mediating T-cell immune responses, its role in UC remains elusive. Although recent studies have shown a strong association between HLA-DRB1 and UC, this association has failed to yield any changes in rates of UC as individual carriers have not been shown to have an increased predisposition to develop UC, a pattern suggestive of external variables contributing significantly to the development of UC in individuals with such genetic variants [18]. Environmental factors and lifestyle modifications have also been implicated in the rising prevalence of UC. Modern lifestyles characterized by increased stress levels, sedentary behavior, and unhealthy dietary choices may contribute to the development or exacerbation of UC [19]. Environmental factors such as air pollution, exposure to certain medications, and changes in the composition of the gut microbiota have also been proposed as potential triggers for UC [20]. The risk of developing UC varies among different populations and ethnic groups. It is more commonly diagnosed in developed countries, suggesting a potential link to industrialization and urbanization [21]. A westernized diet, high in processed ingredients and low in fiber, has been associated with an increased risk of UC. Conversely, populations with traditional diets, rich in fruits, vegetables, and whole grains, exhibit lower rates of UC [22].

In addition to the typical symptoms of UC affecting the colon and rectum, some patients may also experience extra-colonic manifestations, presenting as symptoms beyond the gastrointestinal tract [23]. Acid reflux, nausea, and vomiting are amongst the more commonly reported symptoms [24]. Prior studies have implicated the acute management of UC in exacerbating upper gastrointestinal symptoms when treating for flareups. Corticosteroids are still the first-line choice [25]. Immunomodulator therapies are next in line, typically with TNF-Alpha inhibitors like Infliximab and calcineurin inhibitors like Tacrolimus [25]. These agents can predispose susceptible patient populations to worsening upper gastrointestinal symptoms [26,27], exacerbating the already pro-inflammatory state of the gastrointestinal tract [28]. These symptoms are detrimental to the overall well-being of patients with UC, exacerbating their discomfort and impacting their quality of life. It is imperative for healthcare providers to address and manage these extra-colonic manifestations along with the underlying UC to provide comprehensive care to patients and alleviate their symptoms effectively.

GERD is a common gastrointestinal condition, estimated to impact approximately 20% of the general population. Recognized risk factors predisposing to GERD include tobacco use, obstructive sleep apnea, obesity, and hiatal hernia [29,30]. It is characterized by the regurgitation of the gastric content into the lower esophagus, primarily due to dysfunction of the lower esophageal sphincter. This localized irritation can cause a proinflammatory reaction and induce histological changes with prolonged exposure. In a similar pattern to IBD, the incidence of GERD has experienced a recent surge, a trend that is concurrent with the escalating rates of obesity, changes in dietary habits, and the rise in prevalence of metabolic syndromes [31,32,33]. GERD is commonly encountered clinically with frequently reported symptoms such as nocturnal cough and reflux; these symptoms are indicative of gastric acid regurgitation and local inflammatory response. Endoscopic evaluation and pH-impedance testing are the preferred diagnostic modalities for GERD, capable of distinguishing the characteristic mucosal injuries resulting from prolonged acid exposure [34].

Despite UC and GERD being commonly encountered pathologies in clinical practice, evidence of their potential interrelation remains scarce. Several retrospective studies have discovered an unexpected inverse association between acid reflux and chronic non-infectious colitis, hypothetically driven by chronic gastritis-induced, long-term diminution in gastric acid production compromising the mucosal layer, thereby enhancing susceptibility to bacterial invasion. This process, they proposed, might confer a protective effect against non-infectious colitis [35,36], with recent speculation into the chronic use of proton pump inhibitors (PPIs), potentially exhibiting a similar outcome due to the parallels in their mechanism of action; however, contradictory findings were reported in a more recent study, with evidence of increased rates of UC associated with the concurrent use of PPIs [37].

While acknowledging the existing evidence, which presents an ambiguous and indefinite picture regarding the correlation between UC and GERD, we aim to highlight the potential interdependent relationship between the two conditions. Additionally, we aim to explore the possible interplay between UC and various GERD complications, including esophageal stricture and Barrett’s esophagus (BE), both with and without dysplasia.

## 2. Materials and Methods

### 2.1. Data Source

In our study, we adopted a retrospective framework and made use of the National Inpatient Sample (NIS) database. The NIS is the most comprehensive all-payer inpatient healthcare database accessible to the public, which provides a distinctive avenue for estimating a range of parameters. These include inpatient utilization, accessibility, expenditure, quality, and outcomes. Annually, the database aggregates data from over seven million hospitals, sourced through a representative 20% stratified sampling of discharges from U.S. community hospitals. This database excludes data from rehabilitation and long-term acute care hospitals.

### 2.2. Data Extraction and Outcome Measures

We included a total of 7,159,694 adult hospital-admitted patients. We compared patients diagnosed with GERD (ICD-10-CM K21.9 and K21.0) both with and without UC (ICD-10-CM K51) to those without a GERD diagnosis. We excluded patients with a history of foregut surgeries, uncontrolled type 2 diabetes (T2DM), eosinophilic esophagitis, and infective esophagitis. For a more granular analysis, we examined two GERD phenotypes, non-erosive reflux disease (NERD), and erosive esophagitis (EE).

UC diagnosis was determined based on biopsy or endoscopic evaluation. For those diagnosed with UC, we further sub-stratified our analysis, accounting for pancolitis, left-sided colitis, Rectosigmoiditis, proctitis, and indeterminate UC (patients with an indetermined extent of colonic involvement in the database).

The risk factors of UC (including gender, age, and race), and GERD (including hiatal hernia, cigarette smoking, and obesity) were used for variable adjustment analysis. Patient demographics and comorbidities were collected, including age, race, gender, obesity, controlled T2DM, hiatal hernia, hypertension, and smoking history.

In our study, GERD complications (defined as esophageal stricture and BE, with and without dysplastic changes) were only considered in the group of patients diagnosed with GERD. To evaluate the odds ratio of GERD and its related complications in UC patients, we included patients with GERD and GERD complications (cases) against those without the diagnosis of GERD and GERD complications (controls). All diagnoses included or excluded from this study were selected by the ICD-10-CM code.

### 2.3. Statistical Analysis

In this study, the NIS data comprised categorical information, encompassing demographic details and risk factors, presented as case numbers and percentages. Chi-squared analysis was utilized to assess the association between GERD and UC, as well as to explore GERD complication relationships in patients with and without UC. To quantify GERD risk and its complications concerning distinct types of UC, a multivariate logistic regression analysis calculated the odds ratios, controlling for potential confounders like age, gender, race, cigarette smoking, hiatal hernia, and obesity. A 2-sample test for equal proportions was applied, with a *p*-value below 0.05 indicating statistical significance. All statistical analyses were performed using IBM SPSS software, version 28.0.1.1, ensuring a rigorous analytical approach.

## 3. Results

### 3.1. Demographics

**Figure 1:**
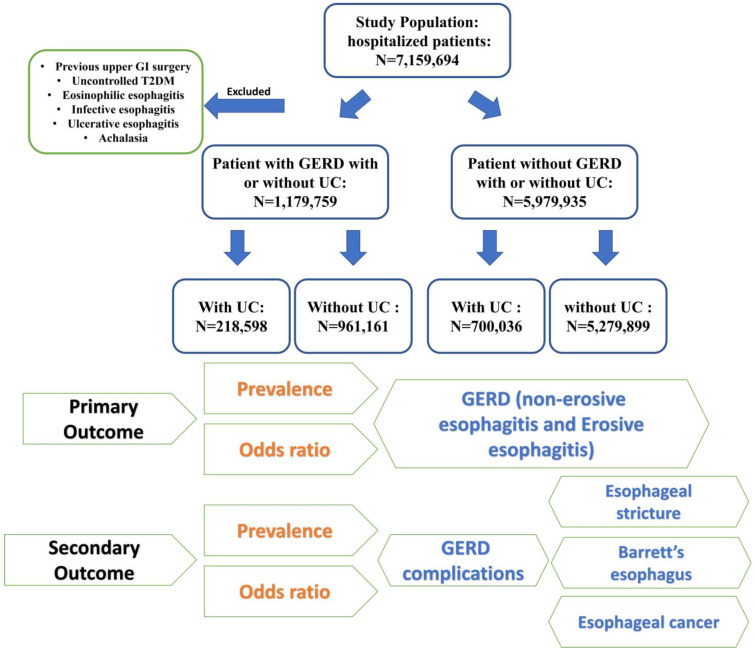
Sample selection and study design flowchart.

Our study incorporated a substantial dataset of 7,159,694 hospital-admitted patients, identifying 1,179,759 patients diagnosed with GERD. Among them, 5796 had UC and 1,173,963 did not. A closer look at the demographic profile showed that patients diagnosed with both GERD and UC were, on average, younger than their counterparts without UC (61.9 ± 0.3 years vs. 64.3 ± 0.2 years, *p* < 0.05) [Figure 1].

### 3.2. Comorbidities

A lower rate of obesity was observed among patients diagnosed with both GERD and UC compared to those without UC (9.9% vs. 14.4%, *p* < 0.01). Additionally, the prevalence of cigarette smoking was lower in the GERD with UC group compared to the GERD without UC group (9.9% vs. 14.4%, *p* < 0.01) [Table 1].

### 3.3. Clinical Outcomes

**Figure 2:**
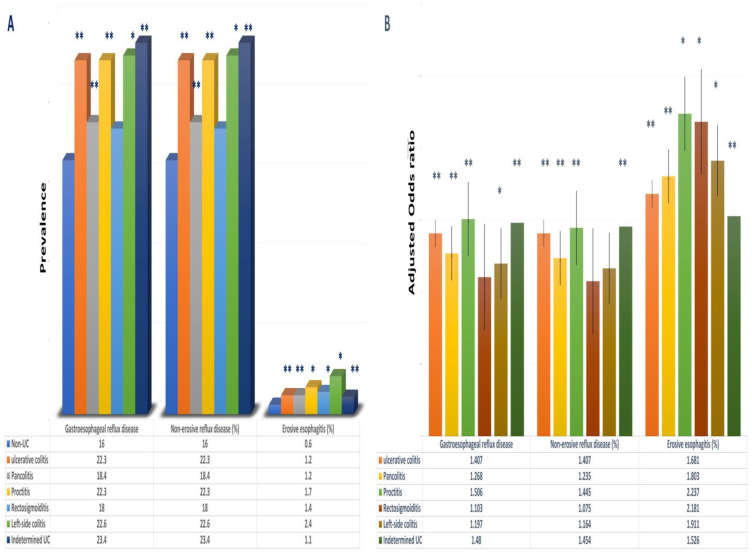
Bar graph of prevalence and odds ratio for patients with UC or subtypes of UC with GERD, NERD, or EE. (**A**) Prevalence of UC and different subtypes of UC with GERD, NERD, and EE. (**B**) Adjusted odds ratio of UC or subtypes of UC with GERD, NERD, or EE. Adjusted for age, sex, race, obesity, hiatal hernia, and history of smoking (* *p* < 0.05); (** *p* < 0.01). Gastroesophageal reflux disease [GERD]; ulcerative colitis [UC]; non-erosive reflux disease [NERD]; erosive esophagitis [EE].

Patients with UC were more likely to have a concurrent diagnosis of GERD than those without UC (23.0% vs. 16.5% *p* < 0.01). These findings were reproducible even when adjusting for potential confounders, thus recognizing UC as a potential independent risk factor for developing GERD (OR: 1.421 95%CI: 1.377–1.466, *p* < 0.01) [Table 2 and Figure 2A].

A sub-analysis of GERD noted that UC patients were significantly more likely to develop NERD compared to those without UC (OR 1.407, 95% CI 1.363–1.453, *p* < 0.01). The incidence of NERD in those with UC was 22.30%, compared to 16.0% in those without UC (*p* < 0.001) [Figure 2B].

The rate of erosive esophagitis (EE) in patients with UC was found to be 1.2%, much higher than that of those without UC at 0.6% (*p* < 0.01) [Figure 2A]. This is indicative of a higher odds of developing EE in patients with UC than those without UC (OR: 1.681, 95%CI: 1.1.467–1.926, *p* < 0.01) [Table 2 and Figure 2B].

Upon further analysis, when accounting for the subtypes of UC in comparison with patients without UC, the incidence of GERD was also found to be elevated in patients with indeterminate UC (23.4%), left-sided colitis (22.6%), ulcerative proctitis (22.3%), and pancolitis (18.4%) (*p* < 0.01) [Figure 2A]. The likelihood of developing GERD was consistently replicated amongst all subtypes, namely, ulcerative proctitis (OR: 1.506, 95%CI: 1.235–1.836, *p* < 0.01), indeterminate UC (OR: 1.480, 95%CI: 1.427–1.534, *p* < 0.01), ulcerative pancolitis (OR: 1.268, 95%CI: 1.166–1.380, *p* < 0.01), and left-sided colitis (OR: 1.197–1.401, *p* < 0.05) [Figure 2B].

A simultaneous rise in the incidence of NERD was observed in patients with UC of different subtypes, including indetermined UC (23.4%), left-sided colitis (22.6%), ulcerative proctitis (22.3%) pancolitis (18.4%), and Rectosigmoiditis (18.0%), significantly higher than those without UC (16%) [Figure 2A]. The corresponding odds of developing NERD, per-UC subtype, compared to patients without UC conformed with these findings, with indeterminate UC (OR: 1.454, 95%CI: 1.402–1.508, *p* < 0.01), ulcerative proctitis (OR: 1.445, 95% CI: 1.181–1.768, *p* < 0.01), and pancolitis (OR: 1.235, 95%CI: 1.133–1.345, *p* < 0.01) [Figure 2B].

The incidence of EE in patients with UC, per subtype, was also consistently elevated, with left-sided colitis (2.4%), ulcerative proctitis (1.7%), Rectosigmoiditis (1.4%), and pancolitis (1.2%), and indetermined UC (1.1%), all of which are significantly higher than that of patients without UC (0.6%) [Figure 2A]. The resultant odds of developing EE followed a similar trend in patients with ulcerative proctitis (OR: 2.237 95%CI: 1.123–4.905), Rectosigmoiditis (OR: 2.181 95%CI: 1.057–3.252), left-sided UC (OR: 1.911, 1.123–3.252), ulcerative pancolitis (OR: 1.803 95%CI: 1.467–1.926), and indetermined UC (OR: 1.526, 95%CI: 1.325–1.841) [Figure 2B].

**Figure 3:**
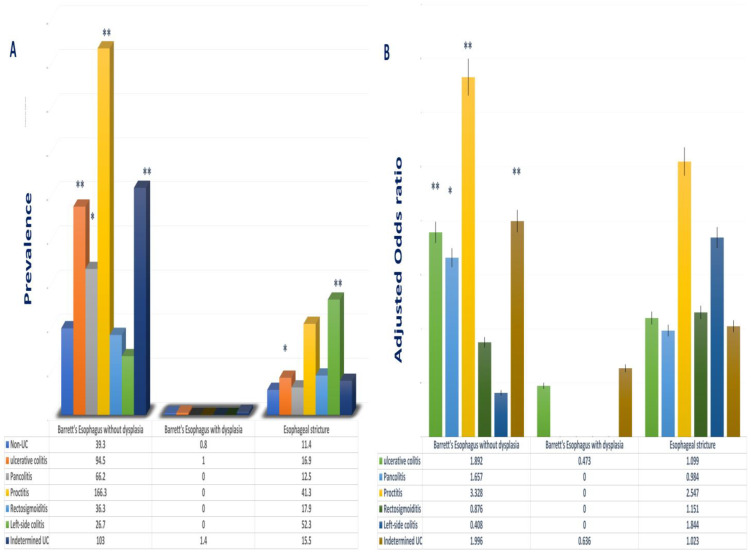
Bar graph of prevalence and odds ratio for patients with UC or subtypes of UC with GERD complications. (**A**) Prevalence of UC and different subtypes of UC with Barrett’s esophagus and esophageal stricture. (**B**) Adjusted odds ratio of UC or subtypes of UC with Barrett’s esophagus and esophageal stricture. GERD, gastroesophageal reflux disease; UC, ulcerative colitis. Adjusted for age, sex, race, obesity, hiatal hernia, and history of smoking (* *p* < 0.05); (** *p* < 0.01).

Among GERD-related complications, the incidence of Barrett’s esophagus without dysplasia in UC was 94.5 per 10,000 patients, compared to that of patients without UC, which was 39.3 per 100,000 (*p* < 0.01). This is reflective of a steep rise in the probability of developing said condition in patients with UC (OR 1.892, 95%CI 1.621–2.209, *p* < 0.001) [Figure 3B]. These findings were further sub-stratified into the different subtypes of UC, ulcerative proctitis (166.3 per 10,000), indetermined UC (103.0 per 10,000), and ulcerative pancolitis (66.2 per 10,000) [Table 2 and Figure 3A].

The odds of patients with UC developing Barrett’s esophagus without dysplasia were comparatively increased in patients with UC, specifically ulcerative proctitis (OR: 3.328, 95%CI: 1.543–7.177), indeterminate UC (OR: 1.996, 95%CI: 1.677–2.376), and ulcerative pancolitis (OR: 1.657 95%CI: 1.070–2.566) [Table 2 and Figure 3B].

The incidence of esophageal strictures in patients with UC was notably higher than that of their counterparts at 16.9 per 10,000 patients, in comparison with 11.4 per 10,000 patients (*p* < 0.05). However, this trend was exclusive to patients with left-sided colitis (52.3 per 10,000 patients) [Table 2 and Figure 3A]. This statistical inclination is likely attributed to the limited sample size of patients with esophageal strictures in the UC cohort compared to those without UC. No additional risk of developing esophagus strictures was denoted in the UC group.

Similarly, and also likely due to the inadequate sample size, there was no statistically significant increase in the prevalence or risk of developing Barrett’s esophagus with dysplasia in patients with UC [Table 2 and Figure 3B].

## 4. Discussion

Our most noteworthy finding over the course of this study was the significantly increased risk and incidence of GERD, along with its related complications, in patients with UC compared to those without UC. This finding was consistent even when adjusting for potential confounding variables such as age, gender, race, hiatal hernia, T2DM, and smoking history. This study represents the inaugural step to leverage a comprehensive inpatient database specifically designed to scrutinize the correlation between UC and GERD. Furthermore, our study unexpectedly unearthed two unforeseen findings while analyzing the potential interrelationship between UC subtypes and GERD. Firstly, there is a heightened risk for a polymorphic presentation of GERD, including NERD and EE, among patients with UC. Secondly, a greater risk and incidence of both NERD and EE were found across all classifications of UC. Thirdly, an increased prevalence and risk of GERD complications, specifically Barrett’s esophagus without dysplasia, were identified in patients with various types of UC.

Research has shown that there is a link between UC and GERD, but the exact nature of this connection is not fully understood. A recent study aimed to unravel this connection by exploring visceral sensitivity in patients diagnosed with UC compared to healthy volunteers (HVs). This investigation was comprised of a modest group, including ten UC patients and twelve HVs. Participants underwent a process of gradual esophageal balloon distension, with concurrent monitoring of their heart rate and blood pressure. The experiment aimed to quantify two main objectives: the air volume required to reach the perceptive threshold and the volume increment necessary to induce pain. The data revealed that the perceptive thresholds of UC patients were similar to those of HVs. In contrast, with regard to pain threshold, UC patients needed a significantly lower volume increment, suggesting a lower pain threshold and a heightened sensitivity to esophageal pain. Upon subjecting the participants to experimental stress through a cold-water pressure test, both cohorts exhibited a decrease in perceptive thresholds. However, the significant reduction in pain thresholds was only observable in HVs. These observations supported the conclusion that UC corresponds with increased esophageal sensitivity, possibly indicative of diffuse hyperalgesia during intestinal inflammatory processes in UC patients, potentially shedding light on the mechanism behind the recurrent upper gastrointestinal symptoms reported by these patients, even in periods of apparent clinical remission [38].

To further our understanding of gastroduodenal involvement in UC patients, a larger scale prospective study was implemented, comprised of 250 UC patients in Japan. In this study, gastroduodenitis associated with ulcerative colitis (GDUC) was determined endoscopically as friable and granular mucosa while clinically excluding other disorders like Crohn’s disease. It was found that the prevalence of GDUC was 7.6% (19 out of 250 patients). Patients with GDUC had a unique set of clinical features, including a more extensive colitis, a lower intake of prednisolone, a higher incidence of pouchitis, and a lengthier postoperative period. This study proved that pancolitis and lower dose of prednisolone were identified as significant individual risk factors for developing GDUC. Thus, the possibility of inflammatory reactions in UC extending beyond the large intestine was underscored. The discrepancy in the prevalence of GDUC suggests a potential risk of underdiagnosis, likely due to the coinciding use of steroids in the treatment of UC, potentially masking its presence [39].

In subsequent studies on the relationship between UC and gastroduodenal involvement, a retrospective study closely examined the statistical trends in the prevalence of, and characteristics of, Ulcerative Gastro-Duodenal Lesions (UGDLs) among UC patients. This study incorporated a sample of 322 patients diagnosed with UC, who had previously undergone upper gastrointestinal endoscopy. UGDL associated with UC was identified by two major criteria. First, a positive diagnosis was established if the lesions demonstrated significant improvement following UC treatment. Secondly, the presence of pathological findings akin to UC substantiated the diagnosis. Out of the total patients examined, 15 were found to features consistent with UGDL, accounting for approximately 4.7% of the total patient cohort. Notably, all 15 patients displayed features of pancolitis or were post-proctocolectomy. This study unveiled a notable discovery concerning the rates of UGDL among patients with UC; post-proctocolectomy patients showed the highest incidence rates at 7.4%, closely followed by patients with pancolitis at 6.2% [40]. These findings agree with several reported cases globally that suggest a link between severe esophageal ulcers and UC [41]. These recurring patterns warrant a thorough research approach to uncover the intricate relationship between UC and other extra-colonic gastrointestinal manifestations. This deeper understanding can pave the way towards more effective and comprehensive therapeutic strategies for UC.

One significant discovery from our study emphasized a greater occurence of Barrett’s esophagus without dysplasia in UC patients compared to those without UC. The more pronounced risk association is particularly notable in patients with ulcerative proctitis (300%), unspecified UC (90%), and ulcerative pancolitis (60%).

Both UC and BE are defined as chronic pro-inflammatory conditions that primarily affect different anatomical areas—the colon and rectum in UC, and the inner esophageal mucosal lining, which in turn undergoes metaplasia into a tissue type resembling that commonly found in the intestine. Recent studies have suggested a potential connection between these two conditions. In a multicenter, retrospective study adopting a propensity score-matching approach, researchers compared the rates of dysplasia, nodular disease, and segment length in patients diagnosed with both BE and inflammatory bowel disease (IBD) to patients with BE alone. Controls were meticulously selected based on variables such as age, sex, BMI, smoking status, and presence of hiatal hernia. The study encompassed a total of 132 patients in both the IBD + BE group and the BE only group. The results revealed that patients with both IBD + BE had higher rates of esophageal dysplasia than controls and a higher prevalence of nodules. Additionally, the IBD + BE group was associated with longer BE segments [42]. These findings may warrant more frequent surveillance for BE, with shorter intervals in individuals co-diagnosed with IBD. Despite this, current guidelines for performing esophagogastroduodenoscopy (EGD) in patients with UC are primarily focused on specific secondary indications rather routine use. According to recent guidelines from the ECCO and ACG, EGD is exclusively reserved for IBD patients with upper gastrointestinal symptoms suggestive of a concurrent disease process, more so in patients with Crohn’s disease [43,44], and some patients with UC due to its association with primary sclerosing cholangitis as well as upper gastrointestinal symptoms attributed to treatment-related side effects in the management of acute flareups with steroid therapy, with most endoscopic interventions focused on lower gastrointestinal complications in patients with UC [45,46,47,48]. Our findings, if validated, would introduce a new indication for screening EGD in patients with UC for surveillance of early upper gastrointestinal features suggestive of GERD and related complications, with a lower threshold for initial EGD and closer surveillance intervals in those experiencing upper gastrointestinal symptoms such as acid reflux or dyspepsia, or a pre-established diagnosis of EGD. This potentially warrants an integrated upper and lower gastrointestinal luminal evaluation with EGD in addition to the current recommendation of colonoscopy in UC patients at the 8th year mark. However, further prospective studies are necessary to validate and reproduce these findings.

Despite these findings, the exact nature of the association between UC and BE remains unclear. It is possible that the two conditions share common risk factors, such as smoking or obesity, which could explain the observed association. Alternatively, UC and BE may be linked through a common mechanism of chronic inflammation, which could promote the development of both conditions.

Oxidative stress, an imbalance between the production of reactive oxygen species (ROS) and the body’s ability to neutralize them, has been implicated in the pathogenesis of various inflammatory diseases. UC, a chronic inflammatory bowel disease, is characterized by oxidative stress-mediated tissue damage in the colon. Several studies suggest that oxidative stress is involved in the severity of ulcerative colitis, and there are alterations in the antioxidant defense system in the erythrocytes of UC patients [49]. A total of 81 adult UC patients and 85 age- and sex-matched apparently healthy controls were included in a study conducted in India. The levels of lipid peroxidation (LPO), reduced glutathione (GSH), catalase, and superoxide dismutase (SOD) were measured in erythrocytes. The levels of LPO, catalase, and SOD in UC patients were found to be significantly increased (*p* < 0.05) compared to healthy controls, while GSH levels in UC patients were significantly decreased [50]. Additionally, oxidative stress has also been reported as an essential mechanism in the pathophysiology of BE. A study examining mucosal specimens from 59 patients with various esophageal conditions showed that oxidative stress increased progressively, indicated by higher myeloperoxidase activity, while antioxidant capacity decreased, as seen in a lower glutathione content. Simultaneously, the formation of DNA adducts indicated DNA damage. Pooled data analysis revealed a negative correlation between glutathione content and DNA adducts. The reflux disease–metaplasia–carcinoma sequence was associated with increased microvasculature and a higher percentage of immature blood vessels [51]. In another study focusing on oxidative stress involving 42 patients with BE and 15 controls, researchers found elevated levels of oxidative stress markers, along with lower levels of GSH in patients with BE, suggesting reduced antioxidant function [52]. There is a possibility that oxidative stress can extend beyond the colon and possibly involve the esophagus, contributing to the pro-inflammatory stimuli driving the progressive histological shift in the development of BE. While additional research is required to validate a direct causal relationship, emerging evidence underscores the potential role of oxidative stress linking UC to BE development. This highlights opportunities for further investigation and potential therapeutic strategies.

We have encountered several limitations to our study. Firstly, despite the comprehensive data provided by the Nationwide Inpatient Database (NIS), our analysis did not account for outpatient therapy and relevant clinical information; we were unable to determine the choice of chronic suppressive therapy, the rate of flareups, and the need and compliance with prophylactic proton pump inhibitor (PPI) treatments for patients requiring intravenous corticosteroid therapy to achieve remission, or those receiving immunomodulator therapy implicated in predisposing patients to upper gastrointestinal adverse events. Therefore, our findings may not be fully reflective of the true impact of GERD and related complications if extrapolated to the general population. Secondly, the diagnosis of GERD and related complications, including esophageal stricture and Barrett’s esophagus, relied solely on the assigned ICD-10-CM codes. These codes are often entered by various hospital personnel and electronic medical records, introducing the possibility of coding errors or inconsistencies. Ideally, the diagnosis of GERD and related complications should be substantiated by additional diagnostic modalities such as imaging, luminal evaluation with EGD, and colonoscopy, as well as histopathological evaluation to ensure consistency and a standardized approach. Moreover, the recognition of common risk factors for GERD, such as smoking, diabetes, and hiatal hernia, also relies on ICD-10-CM codes. However, the timeline of related risk factor exposure is limited by the lack of duration data for related exposure.

To sum up, the lack of outpatient therapeutic and clinical data, unilateral reliance on ICD-10-CM codes for diagnosis and risk factor identification, and the lack of timeline data for these risk factors are crucial variables when interpreting our findings. Future research should aim to address these limitations and provide a more comprehensive understanding of GERD and its associated factors.

## 5. Conclusions

In our study of 7,159,694 hospital-admitted patients, we found significant associations between UC and GERD. UC patients had a higher incidence of GERD, particularly NERD and EE. The risk of NERD and EE was elevated in UC patients, with the highest incidence seen in ulcerative pancolitis, proctitis, left-sided colitis, and indeterminate UC. Barrett’s esophagus without dysplasia, a GERD-related complication, was significantly more prevalent in UC patients, especially those with ulcerative pancolitis, proctitis, and indeterminate UC. Furthermore, a significant rise in the prevalence of esophageal stricture was observed among patients with UC. These findings contribute to our understanding of the GERD–UC relationship, highlighting the need for further research and clinical management.

## Figures and Tables

**Table 1:** Demographics, risk factors, and comorbidities between gastroesophageal reflux disease [GERD] with and without ulcerative colitis [UC].

	GERD w/ UC	GERD w/o UC	*p* Value
Age	61.9 ± 0.3	64.3 ± 0.2	<0.05
Sex			
Female	3401 (58.7%)	686,089 (58.4%)	>0.05
Male	2395 (41.3%)	487,836 (41.6%)	>0.05
Race			
White	4615 (79.6%)	860,522 (73.3%)	<0.05
Black	474 (8.2%)	144,190 (12.3%)	<0.05
Hispanic	296 (5.1%)	81,313 (6.9%)	<0.05
Asian	65 (1.1%)	18,296 (1.6%)	<0.05
Risk factors			
Contr T2DM	594 (10.2%)	150,055 (12.8%)	<0.05
Obesity	986 (17.0%)	237,327 (20.2%)	<0.05
Smoking	575 (9.9%)	169,227 (14.4%)	<0.01
Hiatal Hernia	391 (6.7%)	64,332 (5.5%)	<0.05

**Table 2:** Prevalence and odds ratio for UC patients with GERD and GERD-related complications. Adjusted for age, race, gender, obesity, smoking history, hiatal hernia.

	Gastroesophageal reflux disease					
UC	case	prevalence	*p* value	Adjusted OR	95%CI	*p* value
YES	5796	22.30%	<0.01	1.407	1.363–1.453	<0.01
NO	1,173,963	16.00%				
UCPAN	747	18.40%	<0.01	1.268	1.166–1.380	<0.01
UCPROC	145	22.30%	<0.01	1.506	1.235–1.836	<0.01
UCRECTSIG	128	18.00%	>0.05	1.103	0.903–1.347	>0.05
UCLEFT	236	22.60%	<0.01	1.197	1.023–1.401	<0.05
UCOTHER	4454	23.40%	<0.01	1.48	1.427–1.534	<0.01
	Non-erosive reflux disease					
UC	case	prevalence	*p* value	Adjusted OR	95%CI	*p* value
YES	5563	22.30%	<0.01	1.407	1.363–1.453	<0.01
NO	1,139,480	16.00%				
UCPAN	710	18.40%	<0.01	1.235	1.133–1.345	<0.01
UCPROC	137	22.30%	<0.01	1.445	1.181–1.768	<0.01
UCRECTSIG	121	18.00%	<0.01	1.075	0.877–1.317	>0.05
UCLEFT	218	22.60%	<0.01	1.164	0.992–1.366	>0.05
UCOTHER	4296	23.40%	<0.01	1.454	1.402–1.508	<0.01
	Erosive esophagitis					
UC	case	prevalence	*p* value	Adjusted OR	95%CI	*p* value
YES	238	1.20%	<0.01	1.681	1.467–1.926	<0.01
NO	35,471	0.60%				
UCPAN	37	1.20%	<0.01	1.803	1.288–2.525	<0.01
UCPROC	8	1.70%	<0.01	2.237	1.123–4.905	<0.05
UCRECTSIG	8	1.40%	<0.01	2.181	1.057–4.503	<0.05
UCLEFT	18	2.40%	<0.01	1.911	1.123–3.252	<0.05
UCOTHER	161	1.10%	<0.01	1.526	1.325–1.841	<0.01
	Barrett’s esophagus w/o dysplasia					
UC	case	Case per 10,000	*p* value	Adjusted OR	95%CI	*p* value
YES	184	94.5	<0.01	1.892	1.621–2.209	<0.01
NO	23,522	39.3				
UCPAN	21	66.2	<0.05	1.657	1.070–2.566	<0.05
UCPROC	8	166.3	<0.01	3.328	1.543–7.177	<0.01
UCRECTSIG	2	36.3	>0.05	0.876	0.217–3.535	>0.05
UCLEFT	2	26.7	>0.05	0.408	0.101–1.655	>0.05
UCOTHER	146	103	<0.01	1.996	1.677–2.376	<0.01
	Barrett’s esophagus w/ dysplasia					
UC	case	Case per 10,000	*p* value	Adjusted OR	95%CI	*p* value
YES	2	1	>0.05	0.473	0.066–3.369	>0.05
NO	530	0.8				
UCPAN	0	N/A	N/A	N/A	N/A	N/A
UCPROC	0	N/A	N/A	N/A	N/A	N/A
UCRECTSIG	0	N/A	N/A	N/A	N/A	N/A
UCLEFT	0	N/A	N/A	N/A	N/A	N/A
UCOTHER	2	1.4	>0.05	0.636	0.089–4.531	>0.05
	Esophageal stricture					
UC	case	Case per 10,000	*p* value	Adjusted OR	95%CI	*p* value
YES	33	16.9	<0.05	1.099	0.773–1.561	>0.05
NO	6849	11.4				
UCPAN	4	12.5	>0.05	0.984	0.366–2.645	>0.05
UCPROC	2	41.3	>0.05	2.547	0.614–10.568	>0.05
UCRECTSIG	1	17.9	>0.05	1.151	0.158–8.370	>0.05
UCLEFT	4	52.3	<0.01	1.844	0.672–5.061	>0.05
UCOTHER	22	15.5	>0.05	1.023	0.664–1.576	>0.05

Ulcerative pancolitis [UCPAN]; ulcerative proctitis [UCPROC]; ulcerative Rectosigmoiditis [UCRECTSIG]; left-sided colitis [UCLEFT]; other ulcerative colitis [UCOTHER].

## Data Availability

The data presented in this study are available on request from the corresponding author. The data are not publicly available due to patient and hospital information privacy and the requirement of H.CUP.
